# Characterization of Ultrasound-Extracted *Pouteria splendens* Fruit Extracts: Phytochemical Profiling and Evaluation of Antioxidant and Cytotoxic Effects

**DOI:** 10.3390/foods14060908

**Published:** 2025-03-07

**Authors:** Alejandro Madrid, Valeska Calderón, Valentina Silva, Patricio Novoa, Carlos Jara, Alejandra Catalina Möller, Joan Villena, Cristóbal Balada, Leda Guzmán, Iván Montenegro

**Affiliations:** 1 Laboratorio de Productos Naturales y Síntesis Orgánica (LPNSO), Facultad de Ciencias Naturales y Exactas, Universidad de Playa Ancha, Avda. Leopoldo Carvallo 270, Playa Ancha, Valparaíso 2340000, Chile; valeskacalderonf@gmail.com (V.C.); silvapedrerosv@gmail.com (V.S.); 2 Millennium Nucleus Bioproducts, Genomics and Environmental Microbiology (BioGEM), Avenida España 1680, Valparaíso 2390123, Chile; ivan.montenegro@uv.cl; 3 Herbario del Jardín Botánico Nacional, Viña del Mar 2520000, Chile; pnovoa7@gmail.com; 4 Centro Interdisciplinario de Investigación Biomédica e Ingeniería para la Salud (MEDING), Escuela de Kinesiología, Facultad de Medicina, Universidad de Valparaíso, Valparaíso 2340000, Chile; carlos.jara@uv.cl; 5 Escuela de Tecnología Médica, Facultad de Medicina, Universidad de Valparaíso, Angamos 655, Reñaca, Viña del Mar 2520000, Chile; alejandra.moller@uv.cl; 6 Center of Interdisciplinary Biomedical and Engineering Research for Health (MEDING), Escuela de Medicina, Facultad de Medicina, Universidad de Valparaíso, Angamos 655, Reñaca, Viña del Mar 2520000, Chile; juan.villena@uv.cl; 7 Laboratorio de Química Biológica, Instituto de Química, Facultad de Ciencias, Pontificia Universidad Católica de Valparaíso, Valparaíso 2340025, Chile; cristobal.balada@pucv.cl (C.B.); leda.guzman@pucv.cl (L.G.); 8 Center of Interdisciplinary Biomedical and Engineering Research for Health (MEDING), Escuela de Obstetricia y Puericultura, Facultad de Medicina, Universidad de Valparaíso, Angamos 655, Reñaca, Viña del Mar 2520000, Chile

**Keywords:** *Pouteria splendens*, native fruit, ultrasound-assisted extraction (UAE), polyphenols, HPLC, antioxidant, cytotoxic

## Abstract

This study investigated the bioactive potential of *Pouteria splendens* fruit through ultrasound-assisted extraction, analyzing peel and pulp. The pulp exhibited a higher phytochemical content, with 179.67 mg GAE/g d.w. and 208.48 mg QE/g d.w., approximately 1.5 times more than in the peel. Fifty phenolic compounds were identified by HPLC-MS, including four types of phenolic acids, with hydroxybenzoic (52%) and hydroxycinnamic (42%) as the two predominant ones, and six types of flavonoids, with flavonols (26.67%) and flavones (23.33%) as the two most prevalent. The pulp demonstrated greater antioxidant activity than the peel and the commercial controls (BHA and BHT) in the DPPH assay (IC_50_ 2.54 mg/mL); however, it showed lower activity in the FRAP assay. Cytotoxic activity was evaluated in cancerous (MCF-7, HT-29, and PC-3) and non-cancerous (CCD 841 CoN and HEK-293) cell lines. Notably, the pulp exhibited remarkable cytotoxic activity against colon cancer cells (HT-29), with an IC_50_ of 50 µg/mL, and possible selectivity by not showing significant activity in non-cancerous cells (CoN and HEK). These results suggest that *P. splendens*, and particularly its pulp, is a valuable source of bioactive polyphenols, with potential for the food and pharmaceutical industries.

## 1. Introduction

It is estimated that approximately 10% of all plant species on the planet are edible; however, only 20 different crops provide 90% of the food demand [1]. This has prompted people to use wild fruits to diversify and enrich their modern diets; however, wild fruits have been an integral part of the diet of native people and rural communities since ancient times. The literature highlights the valorization of wild edibles for export and as potential sources of food security, as their use is associated with food scarcity [2]. But one of their remarkable attributes is that they provide a series of compounds beneficial to heath, such as polyphenols [3], which play a vital role in protecting the body against external stimuli and in the elimination of reactive oxygen species (ROS), instigators of various illnesses such as cardiovascular disorders, cancer, diabetes, and neurodegenerative diseases [4]; these ROS-related diseases rank in the top 10 causes of death, accounting for 38% of all deaths in 2021, according to the latest report of the World Health Organization (WHO) [5].

There are wild fruits that, in addition to their culinary appeal, are reported to contain many bioactive compounds, including polyphenols with a great structural diversity and multiple biological effects [6]. An example of this is *Pouteria* species of the Sapotaceae family, which can be widely found in tropical and subtropical regions [7]. Many of them produce high-quality timber and edible fruit, representing a great economic value; an example of this is Lucuma (*Pouteria lucuma*), a characteristic Peruvian fruit, which is estimated to generate revenues of up to USD 10,109,864 for this country [8]. In addition to their commercial importance in the food industry, several species have been used in folk medicine for diverse purposes [9], among them, as an antipyretic, to treat skin affections, digestive disorders, and diabetes [10]. In modern investigations, some biological activities have been reported for species of this genus, such as antioxidant, anti-inflammatory, antibacterial, and antifungal. However, only a few species have been studied so far, among them *Pouteria splendens* (A.DC.) Kuntze.

*Pouteria splendens* (syn. *Gayella valparadisaea* (Molina) Pierre) is an endemic evergreen fruit tree and the only member of the Sapotaceae family in Chile [11]. This tree has a restricted distribution to conservation sites on the Mediterranean coast of central Chile [12], and it is known by the local names “lúcumo silvestre” and “palo colorado”; this species is characterized by producing reddish edible fleshy drupes of 25 to 35 mm in diameter, containing a single rounded seed [13]. According to some ancient texts, Picunches, a native people of central Chile, used the wood of this tree as a building material, its leaves and bark for the treatment of gastrointestinal and joint diseases, and its fruits as a source of food and a base for the manufacture of cakes, jams, and sweets [14]. However, to date no studies have been reported on the phytoconstituents contained in its different parts, only the chemical composition of the oil from the leaves of *P. splendens* is known, in which sesquiterpenes of the aromadendrane and eudesmane type predominate [15], and there are no known studies on the potential biological activities of this tree.

Native medicinal and/or edible fruits are vital to human health, nutrition, and well-being, and research on them is essential to identify and understand the health-promoting metabolites in our food. The benefits derived from their use and the growing interest in the research of plants or fruits containing polyphenols have led to the development of several advanced technologies that allow their extraction without compromising their biological properties to a great extent [16]. Within these methods, the use of ultrasound-assisted extraction is attractive to the food industry [17]. This technology seeks to improve the extraction of components, increase yield, reduce time, and minimize the use of solvents during extraction [18].

Considering that there is support for *Pouteria* fruits as an important source of polyphenols and that *P. splendens* fruits are an endemic medicinal species that lacks previous research, *P. splendens* fruits were chosen for this research because of their potential to reveal new bioactive compounds. The objective of this study was to characterize the phytochemical content and evaluate the antioxidant and cytotoxic activities of extracts obtained by ultrasound-assisted extraction of the peel and pulp of *P. splendens* fruit to investigate and expand the possible applications of this edible fruit as a source of bioactive compounds for the food industry or medical applications.

## 2. Materials and Methods

### 2.1. Reagents

All reagents and solvents were obtained from Aldrich (St. Louis, MO, USA) and were used without further purification.

### 2.2. Plant Material

Fruits of *P. splendens* were manually harvested in February 2024 from the coastal cliffs Acantilados Federico Santa María (33°06′00″ S, 71°44′18″ O), Valparaíso, Chile. A voucher specimen is kept in the Laboratorio de Productos Naturales y Síntesis Orgánica (LPNSO) of the Universidad de Playa Ancha, Valparaíso, Chile (PS-02-24) and was identified by Forest Engineer Patricio Novoa. At the laboratory, the fruits were rinsed with water and then air-dried. After that, the peel was manually separated from the pulp using a knife and then cut into strips, while the pulp was cut into small pieces. The peel and pulp were dried at 60 °C for 14 h using a food dehydrator (Generic, Importadora Montenegro, Santiago, Chile) [19], until the moisture content was 14 ± 3%, and then milled in a grinder to obtain a fine powder that passed through a 100-mesh sieve.

### 2.3. Ultrasound-Assisted Extraction

Ultrasound-assisted extraction was performed according to the method described previously [20,21] with slight modifications using an ultrasonic bath system (TI-H10 MF2 230V, Elma Schmidbauer GmbH, Singen, Germany). An amount of 10 g of dried powder of fruit pulp and peel was mixed with 200 mL of ethanol 96% for 1 h, operating at a constant frequency of 45 kHz, a maximum power of 800 W, and temperature of 60 °C. Cold water was added to maintain a constant temperature (±3 °C) in the ultrasonic bath. Afterwards, the extracts were vacuum-filtered using Whatman No. 1 filter paper and then concentrated to dryness at 40 °C using a vacuum rotary evaporator (Laborota 4001, Heidolph, Schwabach, Germany). Dried extracts of the peel (1.78 g, 17.79% yield) and pulp (2.43 g, 24.3% yield) were stored in amber vials at 4 °C prior to further analysis.

### 2.4. Total Phenolic Content (TPC)

The TPC of extracts was evaluated by the Folin–Ciocalteu technique, and the experimental conditions have been detailed elsewhere [22]. The results were expressed as mg of gallic acid equivalents (GAE) per g of dry weight. Values shown are the mean ± standard deviation of three independent experiments performed in triplicate.

### 2.5. Total Flavonoid Content (TFC)

The TFC of extracts was evaluated using the colorimetric method, and the experimental conditions have been detailed elsewhere [22]. The results were expressed as mg of quercetin (QE) per g of dry weight. Values shown are the mean ± standard deviation of three independent experiments performed in triplicate.

### 2.6. Total Anthraquinone Content (TAC)

The TAC of extracts was evaluated using the colorimetric method, and the experimental conditions have been detailed elsewhere [22]. The results were expressed as mg of emodine (EE) per g of dry weight. Values shown are the mean ± standard deviation of three independent experiments per-formed in triplicate.

### 2.7. Analysis of Phenolic Compounds by High-Performance Liquid Chromatography (HPLC)

The phenolic compounds present in the extracts of *P. splendens* were identified and quantified using an HPLC-1100 Agilent equipped with a 3200 Q TRAP^®^ hybrid quadrupole/linear ion trap mass spectrometer (Sciex, Toronto, Canada). The extracts were reconstituted in 5 mL of methanol and taken to 10 mL with HPLC water. An aliquot was filtered through a 0.45 µm filter, and aliquots of 20 µL were injected in the HPLC system. A 250 × 4.6 mm i.d. 5 µm Nucleosil C18 column was used (operated at 25 °C). The mobile phase consisted of 0.1% formic acid (A) and methanol (100%) (B) at a flow rate of 0.5 mL/min. The elution gradient was 5–50% (B) from 0 to 30 min, followed by an increase to 75% B for 25 min [23]. Mass spectra were obtained over a mass-to-charge ratio (*m*/*z*) of 100–1000 in full scan mode, and the analyses were performed in positive and negative ionization modes. Peak integration and MS spectra acquisition were performed with Thermo XcaliburTM Qualitative Browser (Thermo Xcalibur 2.2 SP1.48, Thermo Fisher Scientific Inc., Waltham, MA, USA).

### 2.8. Antioxidant Activity Test In Vitro

#### 2.8.1. 1,1-Diphenyl-2-picrylhydrazyl (DPPH) Scavenging Assay

To evaluate the antioxidant activity, the protocol established [24] for this methodology was followed, with slight modifications. *P. splendens* extracts and positive controls at different concentrations (0–10 mg/mL) were mixed with a 50 μM DPPH solution in ethanol. A 50 μM DPPH solution (2.9 mL) with ethanol (0.1 mL) was used as the negative control. Butylated hydroxyanisole (BHA) and butylated hydroxytoluene (BHT) were used as positive controls. The extracts and control solutions were incubated for 20 min and their absorbance at 517 nm was measured. Results are presented as mean ± standard deviation from three independent experiments performed in triplicate.

#### 2.8.2. The Ferric Reducing Power (FRAP) Assay

*P. splendens* extracts were tested using the FRAP assay according to the protocol described previously [24]. The assay was performed by mixing freshly prepared FRAP reagent (acetate buffer, TPTZ in hydrochloric acid, and ferric chloride) with deionized water and the sample extract (1.0 mg/mL). The mixture was incubated at 37 °C for 30 min, and absorbance was measured at 593 nm using ethanol as a blank. FRAP values were determined using a Trolox calibration curve and expressed as mM Trolox equivalent antioxidant capacity (mM TEAC). BHT and BHA were used as positive controls. Results are presented as mean ± standard deviation from three independent experiments performed in triplicate.

### 2.9. Cytotoxic Test In Vitro

#### 2.9.1. Cell Lines

Cell lines used in cytotoxicity assays include MCF-7 (human mammary gland adenocarcinoma), HT-29 (human colorectal adenocarcinoma), PC-3 (human prostate adenocarcinoma), CCD 841 CoN (colon epithelial), and HEK-293 (human embryonic kidney) normal cells lines, which were obtained from the American Type Culture Collection (Rockville, MD, USA). All tested cell lines were maintained in a 1:1 mixture of Dulbecco’s modified Eagle’s medium (DMEM) and Ham’s F12 medium, containing 10% heat-inactivated fetal bovine serum (FBS), penicillin (100 U/mL), and streptomycin (100 µg/mL) in a humidified atmosphere with 5% CO_2_ at 37 °C.

#### 2.9.2. In Vitro Growth Inhibition Assay

The cytotoxic activities of extracts were determined using a Sulforhodamine B (SRB) proliferation assay, as described previously [24]. To assess cell viability, cells were seeded at 3·10^3^ cells/well in 96-well plates and incubated at 37 °C with 5% CO_2_. Cells were treated with compounds at concentrations ranging from 12.5 to 250 µg/mL for 72 h. Following treatment, cells were fixed with 10% trichloroacetic acid, stained with 0.1% SRB, and then washed to remove unbound stain. The protein-bound stain was solubilized, and cell density was determined by measuring fluorescence at 540 nm. Daunorubicin and 5-fluorouracil were used as positive controls. Values shown are the mean ± standard deviation of three independent experiments performed in triplicate.

### 2.10. Statistical Analysis

All data are reported as mean values ± standard deviation. Due to non-parametric data, a Kruskal–Wallis ANOVA was used with a confidence level of 95% with STATISTICA 7.0 software.

## 3. Results

### 3.1. Phytochemical Content

Peel and pulp extracts were compared for their total polyphenolic content (TPC), total flavonoid content (TFC), and total anthraquinone content (TAC), as summarized in Table 1.

With respect to the phytoconstituents obtained, the phenol content in the pulp extract was almost 1.6 times higher than that in the peel extract. In addition, pulp also contained slightly more than 1.5 times more flavonoids than peel, and for anthraquinones in the pulp and peel extracts, no significant differences were detected between pulp and peel. These results, in general, differ from studies carried out on edible fruits, where the highest quantification of phytoconstituents is found in the peel. However, this result can be varied by modifying the extraction time, concentration solvent, method, and temperature [25]. Comparing the results obtained with those reported in the literature, the total phenolic content of the pulp of *P. splendens* is 256 times higher than that reported for the Montero biotype and agrees with the content of the other commercial biotypes of *P. lucuma* (Leiva 1 and Rosalia) tested [26]. While studies on *P. campechiana*, a species widely distributed in tropical and subtropical regions of South America and Asia, report values of 2.05 mg GAE/g and 9.30 mg QE/g in pulp, and 1.90 mg GAE/g and 4.80 mg QE/g in peel, both the TPC and TFC of *P. splendens* pulp are notably higher. As for peel, although another study in *P. campechiana* reported values of 39.45 mg GAE/g d.w. and 12.56 mg QE/g d.w. [6], the TPC and TFC of *P. splendens* peel still exceed these values. In another report, the methanolic extract of the pulp of the curriola fruit (*P. ramiflora*), a medicinal plant from Brazil, presented a TPC value (34.2 (GAE)/g) 5.2 times lower than that of the *P. splendens* extract, adding to what was reported for the ultrasound-assisted extraction of mamey pulp (*P. sapota*), which presented a TPC value between 13.4 and 36.0 mg GAE/g [27], well below what was observed for *P. splendens*. On the other hand, the fruits of *P. splendens* showed a higher TPC compared to the peel of other wild edible fruits such as white pitahaya (15.94 mg GAE/g dry weight) and Goji berry (9.2 mg GAE/g dry weight), approximately 7 times [28] and 12 times higher [29], respectively. Many studies have linked the antioxidant, antimicrobial, and anti-cancer activity of various plants and fruits with their phenolic content [30].

### 3.2. Identification of Phenols by HPLC

**Figure 1:**
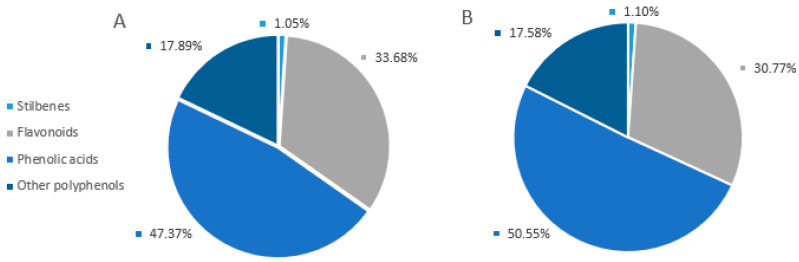
Percentage relative distribution of polyphenols present in the extracts of *P. splendens* peel (**A**) and pulp (**B**).

Several studies indicate that a high intake of fruits invariably leads to a reduced risk of chronic diseases [31,32]. In this sense, knowing the composition of phenolic compounds present in native fruits is becoming increasingly important due to their potential application in the treatment of diseases. In this regard, the presence of many bioactive compounds, such as polyphenolic antioxidants, including gallic acid, catechin derivatives, and flavonoids, has been determined in fruits of approximately 9 of the 325 existing species of *Pouteria* [6]; however, the content of bioactive compounds in fruit extracts of *P. splendens* has not been studied to date. For this reason, this study includes the identification of the phenolic acids, flavonoids, stilbenes, and other polyphenols of *P. splendens* fruit using the RP-HPLC/MS technique. The identification of each peak was possible by retention time and molecular weights, with more than 1200 molecular weights found in the database Phenol-Explorer [33]. In both extracts, it is observed that there is a higher percentage relative distribution of phenolic acids, followed by flavonoids, then other polyphenols, and then stilbenes (Figure 1).

Tables S1–S7 (see Supplementary Material) list the compounds found in both extracts in positive and negative mode, organized according to polyphenol class. In these tables, 50 compounds were identified, distributed in four subclasses of phenolic acids as follows: 52% hydroxybenzoic, 42% hydroxycinnamic, 4% hydroxyphenylpropanoic, and 2% hydroxyphenylacetic acids, represented by biologically active molecules such as gallic acid, chicoric acid, homovanillic acid, and dihydrocaffeic acid, respectively. The following polyphenolic compounds were flavonoids, identifying 30 compounds divided into flavonols with 26.67%, flavones with 23.33%, anthocyanins with 20%, isoflavonoids with 13.33%, flavanones with 10%, and chalcones with 6.67% abundance. Some representatives of these subclasses are kaempferol, 6-hydroxyluteolin, cyanidin, glycitein, naringenin, and butein, respectively. Other important phenolic compounds, such as 4-hydroxycoumarin, syringaldehyde, carnosic acid, 3,4-dihydroxyphenylglycol, guiacol, 1,4-naphthoquinone, and various catechols, were also found. Finally, among the percentage of stilbenes, the presence of resveratrol was reported.

### 3.3. Antioxidant Activity

The antioxidant activity of *P. splendens* peel and pulp extracts was evaluated in two *in vitro* tests, corresponding to DPPH and FRAP. The results are given in Table 2, which shows differences between the antioxidant capacity of peel and pulp extracts; these differences may be due to the higher content of TPC, TFC, and TAC in the pulp compared to the peel, as described in the above.

The DPPH assay is a reliable method for evaluating antioxidant capacity, measuring a substance’s ability to neutralize the DPPH radical. In this study, the peel extract exhibited a higher IC_50_ value than the positive controls in DPPH scavenging activity, indicating the lowest antioxidant activity. This could be attributed to the presence of lipids, proteins, ash, crude fiber, and carbohydrates, or to a lower concentration of phenolic compounds. In contrast, the pulp extract demonstrated a greater capacity to scavenge free radicals compared to the peel extract, suggesting a higher concentration of phenols and flavonoids, consistent with the data presented in Table 1. Notably, the free radical scavenging capacity of the *P. splendens* pulp extract was comparable to, and even superior to, that of common synthetic antioxidants in the food industry such as butylated hydroxytoluene (BHT) and butylated hydroxyanisole (BHA). This antioxidant behavior is consistent with studies conducted on the fruits of other *Pouteria* species, such as lucuma amazonica, *P. macrophylla* and *P. campechiana*, which show the necessary capacity to eliminate radicals using the DPPH assay [34]. Also reported within the scarce antioxidant data are the aqueous extracts of the pulp of *P. glomerata*, which yielded IC_50_ values between 6.41 and 40.71 mmol TE/100 g for this assay [35].

The FRAP assay provides an overall measure of the antioxidant capacity of the sample by evaluating its ability to reduce iron (III) to iron (II). For this case, the FRAP values of both extracts were lower than those obtained by the positive control, and their activity was even lower, 100 times lower, if we compare it with the value obtained by *P. lucuma* in the only FRAP study reported on a fruit of the *Pouteria* genus [36]. However, this assay is useful because it reflects the capacity of the sample to donate electrons and act as a reducing agent in an oxidative environment. Both assays are valuable for evaluating the antioxidant capacity of the samples and can complement each other in order to validate their potential use as a source of bioactive metabolites for the nutraceutical industry.

### 3.4. Cytotoxicity Activity

In recent decades, it has been identified that a low consumption of fruit and vegetables predisposes to the development of a number of cancers, mainly in the colon, where 43% of cases are attributed to this habit [37]. In turn, recent findings support that a higher intake of fruits and vegetables, and specifically yellow/orange vegetables and fruits, may reduce the risk of breast cancer, especially cases that are more likely to be aggressive tumors [38], and some studies suggest that a high consumption of cruciferous vegetables is associated with a lower risk of prostate cancer [39]. For this reason, it is interesting to evaluate the cytotoxic activity against tumor lines of fruit extracts, discarded ripe fruits, or their peel to support the basis for the development of food products or by-products. The cytotoxic activity of peel and pulp extracts of *P. splendens* is presented in Table 3.

The results obtained showed the highest cytotoxic effect on colon cancer cells (HT-29), with better IC_50_ values than against breast (MCF-7) and prostate (PC-3) cell lines. There are few reports for fruits of the genus *Pouteria* on cytotoxic effects on cancer cell lines, for example, the use of ethanol extracts of *P. caimito* fruit for the synthesis of biogenic silver nanoparticles (BAg) and core–shell nanospheres (BAg@SiO_2_) to combat oral cancer [40]; however, in this study the fruit itself was not used to combat the disease.

Previous reports of the activity of *P. sapota* leaves using the MTT assay in breast cancer cells (MCF-7) exposed their cytotoxic capacity, showing changes in cell morphology at concentrations between 25 µg and 125 µg [41]. Regarding the mechanisms of cell death, they have been studied in *P. ramiflora*, which presents antiproliferative effects in the human hepatocellular carcinoma cell line (HepG2); it was demonstrated that extracts of this species caused cycle arrest in S and G2/M phase and induced death by apoptosis [42]. Selectivity is a desirable characteristic for any compound with chemotherapeutic potential; although toxicity in normal cells has not been explored, other studies in *P. ramiflora* and *P. torta* showed no toxicity in *Artemia salina* [43].

In relation to the selectivity of the sample, that is, to the capacity of the cytotoxic agent to specifically attack abnormal cells, minimizing the damage to normal cells, it can be used as an important tool for the selection of samples with potential anti-cancer application; for the case of the samples in this study, they did not show significant activity in the CoN and HEK cell lines, so they could have selectivity, except for the case of the fruit peel in PC-3, which did not show activity in this cancerous line.

Finally, there is a correlation between the cytotoxic activity of the samples and their abundance of phenolic compounds. *P. splendens* pulp showed cytotoxic activity against human colorectal adenocarcinoma cells, directly related to the fact that the pulp extract contains a number of phenols and flavonoids (Table 1) and antioxidant capacity (Table 2). As mentioned, predominant phenols such as gallic acid are found in its composition, consistent with other *Pouteria* spp. This compound is an intermediate in the synthesis of gallotatins, which are important because they are determinants in the antitumor activity of fruit extracts [44]. Gallic acid also stands out for being selectively cytotoxic in different tumor cell lines, including the HT-29 cell line, in which *P. splendens* fruits were also shown to be cytotoxic. The capacity of this compound to inhibit the growth of cancer cells involves multiple molecular targets, with one significant mechanism being associated with the induction of apoptosis [45]. In addition, a variety of molecules belonging to the flavonoid family with anti-cancer potential were identified in this fruit, including kaempferol, 6-hydroxyluteolin, cyanidin, glycitein, naringenin, and butein. Kaempferol, a tetrahi-droxyflavone, acts against various tumors through multiple mechanisms [46]. 6-hydroxyluteolin, a pentahydroxyflavone, shows antiproliferative activity against breast cancer cells, making these fruits a potential dietary source of this molecule, normally present in medicinal plants [47]. Glycitein, an isoflavone, inhibits the proliferation of breast and prostate cancer cells [48]. Cyanidin, an anthocyanin, has antioxidant and anti-inflammatory effects, with the potential to inhibit growth, induce apoptosis, and reduce metastasis in various types of cancer [49]. Naringenin induces apoptosis and cell cycle arrest in several cancer cell lines, including breast, hepatocellular, and prostate cells [50]. Finally, butein, a natural chalcone, shows promising antitumor effects in colorectal cancer [51]. Regarding the presence of stilbenes, these have aroused interest due to their multiple properties, antioxidant, anti-inflammatory, antiangiogenic, among others; within this group, resveratrol stands out for its excellent potential in the prevention and treatment of cancer, being able to inhibit all stages of carcinogenesis, and is also found in extracts of the fruit of *P. splendens* [52]. Taken together, the presence of these bioactive compounds gives the fruit potential value in the prevention and treatment of cancer.

## 4. Conclusions

In conclusion, this initial study reveals that the pulp of *Pouteria splendens*, an endemic Chilean fruit, is a rich source of bioactive polyphenols, exhibiting remarkable antioxidant activity and selective cytotoxic activity against colon cancer cells. The identification of specific phenolic compounds such as gallic acid, kaempferol, and resveratrol, and the demonstration of antioxidant capacity comparable to synthetic antioxidants, suggest the potential of *P. splendens* as a functional ingredient with anti-cancer properties. These findings warrant further investigation into the mechanisms of the action, safety, and efficacy *of P. splendens* and its bioactive constituents, including an exploration of different extraction methods, for the development of food products or by-products with preventive and therapeutic potential against cancer.

## Figures and Tables

**Table 1:** Total phenolic, flavonoid, and anthraquinone contents of *P. splendens* peel and pulp extracts.

Sample	TPC (mg GAE/g d.w.) ^1^	TFC (mg QE/g d.w.) ^2^	TAC (mg EE/g d.w.) ^3^
Peel	112.36 ± 2.97 ^a^	134.68 ± 1.23 ^a^	72.5 ± 2.64 ^a^
Pulp	179.67 ± 0.62 ^b^	208.48 ± 2.59 ^b^	64.49 ± 3.05 ^a^

^1^ mg GAE/g d.w.: mg of gallic acid equivalents per g of dry weight, ^2^ mg QE/g d.w.: mg of quercetin per g of dry weight, ^3^ EE/g d.w.: mg of emodin equivalents per g of dry weight; values expressed as the mean values ± standard deviation of three independent experiments, each performed in triplicate. Different letters in the same column indicate significant differences; *p* < 0.05.

**Table 2:** Antioxidant capacity of the peel and pulp of *P. splendens* extracts given by DPPH and FRAP assays.

Sample	DPPH• IC_50_ (mg/mL)	FRAP TEAC (mM)
Peel	6.73 ± 0.53 ^a^	0.024 ± 0.001 ^a^
Pulp	2.54 ± 0.13 ^b^	0.012 ± 0.005 ^b^
BHT	3.04 ± 0.01 ^c^	1.72 ± 0.01 ^c^
BHA	2.86 ± 0.04 ^d^	1.67 ± 0.03 ^c^

Values expressed as the mean values ± standard deviation of three independent experiments, each performed in triplicate. Different letters in the same column indicate significant differences; *p* < 0.05.

**Table 3:** Cytotoxicity of peel and pulp of *P. splendens* extracts against cancer and non-tumoral cells.

	IC_50_ (µg/mL) ^a^				
Sample	MCF-7	HT-29	PC-3	CoN	HEK
Pulp	100 ± 0.06	50 ± 0.01	200 ± 0.01	>250	>250
Peel	150 ± 0.06	75 ± 0.02	250 ± 0.01	>250	>250
Dau	0.33 ± 0.02	15.11 ± 0.5	0.41 ± 0.04	13.9 ± 0.3	14.3 ± 1.85
5-FU	22.3 ± 0.7	2.9 ± 0.7	16.42 ± 0.6	56.1 ± 0.5	62.5 ± 1.02

^a^ IC_50_ was evaluated using the SRB assay and ± is the standard deviation from three independent experiments.

## Data Availability

The original contributions presented in the study are included in the article/Supplementary Material, further inquiries can be directed to the corresponding author.

## Supplementary Materials

The following supporting information can be downloaded at https://www.mdpi.com/article/10.3390/foods14060908/s1, Figure S1. Calibration curve and correlation factor for total phenol quantification. Figure S2. Calibration curve and correlation factor for total flavonoid quantification. Figure S3. Calibration curve and correlation factor for total anthraquinone quantification. Table S1. Phenolic acids found in peel and pulp extracts in positive mode. Table S2. Phenolic acids found in peel and pulp extracts in negative mode. Table S3. Flavonoids found in peel and pulp extracts in positive mode. Table S4. Flavonoids found in peel and pulp extracts in negative mode. Table S5. Stilbenes found in peel and pulp extracts in positive mode. Table S6. Other polyphenols found in peel and pulp extracts in positive mode. Table S7. Other polyphenols found in peel and pulp extracts in negative mode.
