# Supplementary material for: Characterization of Ultrasound-Extracted Pouteria splendens Fruit Extracts: Phytochemical Profiling and Evaluation of Antioxidant and Cytotoxic Effects
Source: Foods. 2025 Mar 7;14(6):908. doi: 10.3390/foods14060908 (PMC11941558; doi:10.3390/foods14060908)
Supplement: Supplementary file 1 [file foods-14-00908-s001.zip › foods-3497191-supplementary.pdf]

Supplementary Material

Characterization of Ultrasound-Extracted *Pouteria splendens* Fruit extracts: Phytochemical Profiling and Evaluation of Antioxidant and Cytotoxic Effects

Alejandro Madrid [1,2,*], Valeska Calderón [1], Valentina Silva [1], Patricio Novoa [3], Carlos Jara [4], Alejandra Catalina Möller [5], Joan Villena [6], Cristóbal Balada [7], Leda Guzmán [7], Iván Montenegro [2,8]

[1] Laboratorio de Productos Naturales y Síntesis Orgánica (LPNSO), Facultad de Ciencias Naturales y Exactas, Universidad de Playa Ancha, Avda. Leopoldo Carvallo 270, Playa Ancha, Valparaíso 2340000, Chile; valeskacalderonf@gmail.com (V.C.); silvapedrerosv@gmail.com (V.S.)

[2] Millennium Nucleus Bioproducts, Genomics and Environmental Microbiology (BioGEM), Avenida España 1680, Valparaíso 2390123, Chile; ivan.montenegro@uv.cl

[3] Herbario del Jardín Botánico Nacional, Viña del Mar 2520000, Chile; pnovoa7@gmail.com

[4] Centro Interdisciplinario de Investigación Biomédica e Ingeniería para la Salud (MEDING), Escuela de Kinesiología, Facultad de Medicina, Universidad de Valparaíso, Valparaíso 2340000, Chile; carlos.jara@uv.cl

[5] Escuela de Tecnología Médica, Facultad de Medicina, Universidad de Valparaíso, Angamos 655, Reñaca, Viña del Mar 2520000, Chile; alejandra.moller@uv.cl

[6] Center of Interdisciplinary Biomedical and Engineering Research for Health (MEDING), Escuela de Medicina, Facultad de Medicina, Universidad de Valparaíso, Angamos 655, Reñaca, Viña del Mar 2520000, Chile; juan.villena@uv.cl

[7] Laboratorio de Química Biológica, Instituto de Química, Facultad de Ciencias, Pontificia Universidad Católica de Valparaíso, Valparaíso 2340025, Chile; cristobal.balada@pucv.cl (C.B.); leda.guzman@pucv.cl (L.G.)

[8] Center of Interdisciplinary Biomedical and Engineering Research for Health (MEDING), Escuela de Obstetricia y Puericultura, Facultad de Medicina, Universidad de Valparaíso, Angamos 655, Reñaca, Viña del Mar 2520000, Chile

*Correspondence: alejandro.madrid@upla.cl; Tel.: +56-032-250-0526

Index.

Figure S1. Calibration curve and correlation factor for total phenol quantification.

Figure S2. Calibration curve and correlation factor for total flavonoid quantification.

Figure S3. Calibration curve and correlation factor for total anthraquinone quantification

Table S1. Phenolic acids found in peel and pulp extracts in positive mode.

Table S2. Phenolic acids found in peel and pulp extracts in negative mode.

Table S3. Flavonoids found in peel and pulp extracts in positive mode.

Table S4. Flavonoids found in peel and pulp extracts in negative mode.

Table S5. Stilbenes found in peel and pulp extracts in positive mode.

Table S6. Other polyphenols found in peel and pulp extracts in positive mode.

Table S7. Other polyphenols found in peel and pulp extracts in negative mode.

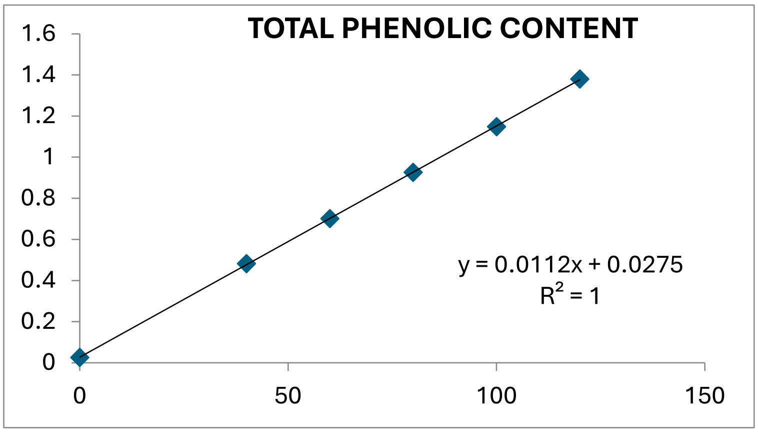

Figure S1. Calibration curve and correlation factor for total phenolic quantification.

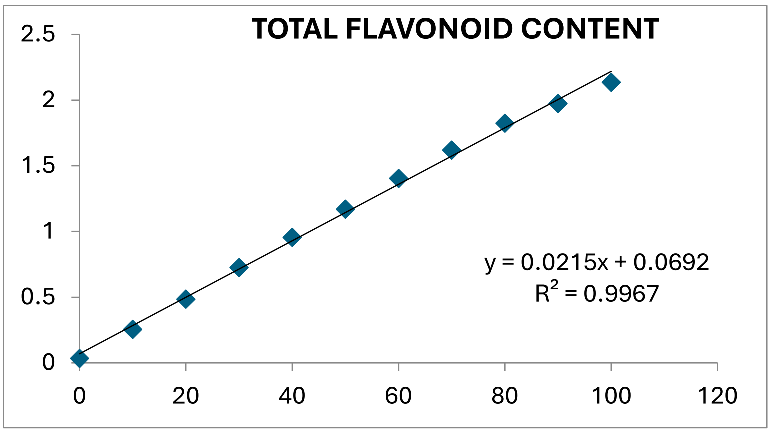

Figure S2. Calibration curve and correlation factor for total flavonoid quantification.

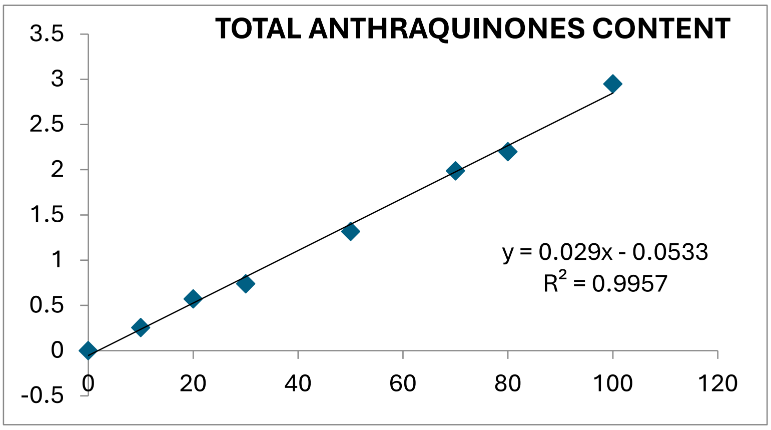

Figure S3. Calibration curve and correlation factor for total anthraquinone quantification

Table S1. Phenolic acids found in peel and pulp extracts in positive mode.

Phenolic acid	Molecular formula	Subclass	Expected mass (m/z)	Expected RT (s)	Peel				Pulp			
					RT Observed (s)	Variation RT (s)	Observed mass (m/z)	Mass variation (m/z)	RT Observed (s)	Variation RT (s)	Observed mass (m/z)	Mass variation (m/z)
3,4-Dihydroxyphenylacetic Acid	$C_8H_8O_4$	Hydroxyphenylacetic Acid	168.15	12.45	12.3	0.15	168.15	0	12.7	-0.25	168.15	0
4-Hydroxybenzoic Acid	$C_7H_6O_3$	Hydroxybenzoic Acid	138.12	13.87	NF	NF	NF	NF	13.94	-0.07	152.15	-14.03
Dihydrocaffeic Acid	$C_9H_{10}O_4$	Hydroxyphenylpropanoic Acid	182.17	18.69	18.79	-0.1	182.16	0.01	18.53	0.16	182.16	0.01
Homovanillic Acid	$C_9H_{10}O_4$	Hydroxyphenylacetic Acid	182.17	18.69	18.79	-0.1	182.16	0.01	18.53	0.16	182.16	0.01
Caffeic Acid	$C_9H_8O_4$	Hydroxycinnamic Acid	180.16	18.75	18.93	-0.18	180.15	0.01	18.55	0.2	180.15	0.01
Gallic Acid 4-O-Glucoside	$C_{13}H_{16}O_{10}$	Hydroxybenzoic Acid	332.26	20.92	20.68	0.24	332.25	0.01	NF	NF	NF	NF
Galloyl Glucose	$C_{13}H_{16}O_{10}$	Hydroxybenzoic Acid	332.26	20.92	20.68	0.24	332.25	0.01	NF	NF	NF	NF
Avenanthramide 2C	$C_{16}H_{13}NO_6$	Hydroxycinnamic Acid	315.28	21.46	NF	NF	NF	NF	21.68	-0.22	315.27	0.01
Avenanthramide K	$C_{16}H_{13}NO_6$	Hydroxycinnamic Acid	315.28	21.46	NF	NF	NF	NF	21.68	-0.22	315.27	0.01
Ellagic Acid Glucoside	$C_{20}H_{16}O_{13}$	Hydroxybenzoic Acid	464.34	22.52	22.37	0.15	464.32	0.02	22.69	-0.17	464.32	0.02
P-Coumaroyl Malic Acid	$C_{13}H_{12}O_7$	Hydroxycinnamic Acid	280.23	22.56	22.61	-0.05	280.23	0	22.61	-0.05	280.24	-0.01

P-Coumaric Acid	$C_9H_8O_3$	Hydroxycinnamic Acid	164.16	22.67	22.65	0.02	164.15	0.01	22.63	0.04	164.15	0.01
Ellagic Acid Arabinoside	$C_{19}H_{14}O_{12}$	Hydroxybenzoic Acid	434.31	22.7	22.62	0.08	434.31	0	22.47	0.23	434.31	0
5-O-Galloylquinic Acid	$C_{14}H_{16}O_{10}$	Hydroxybenzoic Acid	344.27	22.93	23.11	-0.18	344.28	-0.01	23.11	-0.18	344.28	-0.01
Protocatechuic Acid 4-O-Glucoside	$C_{13}H_{16}O_9$	Hydroxybenzoic Acid	316.26	22.93	23.11	-0.18	316.26	0	22.94	-0.01	316.26	0
P-Coumaroyl Tartaric Acid	$C_{13}H_{12}O_8$	Hydroxycinnamic Acid	296.23	23.55	23.44	0.11	296.23	0	23.42	0.13	296.23	0
Salicylic Acid	$C_7H_6O_3$	Hydroxybenzoic Acid	138.12	23.56	23.54	0.02	138.13	-0.01	23.48	0.08	138.13	-0.01
2-Hydroxybenzoic	$C_7H_6O_3$	Hydroxybenzoic Acid	138.12	23.56	23.54	0.02	138.13	-0.01	23.48	0.08	138.13	-0.01
3-Hydroxybenzoic Acid	$C_7H_6O_3$	Hydroxybenzoic Acid	138.12	23.56	23.54	0.02	138.13	-0.01	23.48	0.08	138.13	-0.01
4-Hydroxybenzoic Acid	$C_7H_6O_3$	Hydroxybenzoic Acid	138.12	23.56	23.54	0.02	138.13	-0.01	23.48	0.08	138.13	-0.01
Vanillic Acid	$C_8H_8O_4$	Hydroxybenzoic Acid	168.14	23.84	23.86	-0.02	168.15	-0.01	23.86	-0.02	168.15	-0.01
Cafeoyl Tartaric Acid	$C_{13}H_{12}O_9$	Hydroxycinnamic Acid	312.23	23.85	23.73	0.12	312.24	-0.01	23.68	0.17	312.22	0.01
Sinapic Acid	$C_{11}H_{12}O_5$	Hydroxycinnamic Acid	224.21	23.91	23.71	0.2	224.21	0	23.85	0.06	224.21	0
Gallic Acid 3-O-Gallate	$C_{14}H_{10}O_9$	Hydroxybenzoic Acid	322.22	24.05	23.91	0.14	322.22	0	23.98	0.07	322.22	0
8,5'-Deshidrodiferulic Acid	$C_{20}H_{18}O_8$	Hydroxycinnamic Acid	386.35	24.26	NF	NF	NF	NF	24.1	0.16	386.35	0

8,5'-Benzofuran Diferulic Acid	$C_{20}H_{18}O_8$	Hydroxycinnamic Acid	386.1	24.26	NF	NF	NF	NF	24.1	0.16	386.35	-0.25
5,8'-Deshidrodiferulic Acid	$C_{20}H_{18}O_8$	Hydroxycinnamic Acid	386.35	24.26	NF	NF	NF	NF	24.1	0.16	386.35	0
8-O-4'-Deshidrodiferulic Acid	$C_{20}H_{18}O_8$	Hydroxycinnamic Acid	386.35	24.26	NF	NF	NF	NF	24.1	0.16	386.35	0
4-Hydroxybenzoic Acid 4-O-Glucoside	$C_{13}H_{16}O_8$	Hydroxybenzoic Acid	300.26	25.42	25.66	-0.24	300.26	0	25.65	-0.23	300.26	0
Ellagic Acid Acetyl Arabinoside	$C_{21}H_{16}O_{13}$	Hydroxybenzoic Acid	476.34	25.59	25.6	-0.01	476.34	0	NF	NF	NF	NF
Acetyl-Xyloside of Ellagic Acid	$C_{21}H_{16}O_{13}$	Hydroxybenzoic Acid	476.34	25.59	25.6	-0.01	476.34	0	NF	NF	NF	NF
Cafeoyl Aspartic Acid	$C_{13}H_{13}NO_7$	Hydroxycinnamic Acid	295.24	26.37	26.13	0.24	295.25	-0.01	NF	NF	NF	NF
Hydrocaffeic Acid	$C_9H_8O_5$	Hydroxycinnamic Acid	196.16	26.42	26.25	0.17	196.15	0.01	26.4	0.02	196.15	0.01
Gallic Acid	$C_7H_6O_5$	Hydroxybenzoic Acid	170.12	26.46	26.55	-0.09	170.12	0	26.22	0.24	170.12	0
Ellagic Acid	$C_{14}H_6O_8$	Hydroxybenzoic Acid	302.19	26.55	26.53	0.02	302.2	-0.01	26.3	0.25	302.2	-0.01
Avenanthramide 2P	$C_{16}H_{13}NO_5$	Hydroxycinnamic Acid	299.28	26.61	26.73	-0.12	299.27	0.01	26.57	0.04	299.27	0.01
Caffeic Acid 4-O-Glucoside	$C_{15}H_{18}O_9$	Hydroxycinnamic Acid	342.29	26.71	26.78	-0.07	342.31	-0.02	26.79	-0.08	342.31	-0.02
Coumaroyl Glucose	$C_{15}H_{18}O_9$	Hydroxycinnamic Acid	342.29	26.71	26.78	-0.07	342.31	-0.02	26.79	-0.08	342.31	-0.02
4-O-Glucoside of P-Coumaric Acid	$C_{15}H_{18}O_8$	Hydroxycinnamic Acid	326.29	27.32	27.36	-0.04	326.29	0	27.26	0.06	326.31	-0.02

P-Coumaroyl Glucose	$C_{15}H_{18}O_8$	Hydroxycinnamic Acid	326.29	27.32	27.36	-0.04	326.29	0	27.26	0.06	326.31	-0.02
P-Coumaroylquinic Acid	$C_{16}H_{18}O_8$	Hydroxycinnamic Acid	338.31	27.73	27.61	0.12	338.31	0	27.62	0.11	338.31	0
Chicoric Acid	$C_{22}H_{18}O_{12}$	Hydroxycinnamic Acid	474.37	28.8	28.76	0.04	474.38	-0.01	NF	NF	NF	NF
2,3-Dihydroxybenzoic Acid	$C_7H_6O_4$	Hydroxybenzoic Acid	154.12	35.26	35.35	-0.09	154.12	0	35.16	0.1	154.12	0
2,4-Dihydroxybenzoic Acid	$C_7H_6O_4$	Hydroxybenzoic Acid	154.12	35.26	35.35	-0.09	154.12	0	35.16	0.1	154.12	0
2,6-Dihydroxybenzoic Acid	$C_7H_6O_4$	Hydroxybenzoic Acid	154.12	35.26	35.35	-0.09	154.12	0	35.16	0.1	154.12	0
2,5-Dihydroxybenzoic Acid	$C_7H_6O_4$	Hydroxybenzoic Acid	154.12	35.26	35.35	-0.09	154.12	0	35.16	0.1	154.12	0
Gentisic Acid	$C_7H_6O_4$	Hydroxybenzoic Acid	154.12	35.26	35.35	-0.09	154.12	0	35.16	0.1	154.12	0
Hypogallic Acid	$C_7H_6O_4$	Hydroxybenzoic Acid	154.12	35.26	35.35	-0.09	154.12	0	35.16	0.1	154.12	0
Protocatechuic Acid	$C_7H_6O_4$	Hydroxybenzoic Acid	154.12	35.26	35.35	-0.09	154.12	0	35.16	0.1	154.12	0
Resorcylic Acid	$C_7H_6O_4$	Hydroxybenzoic Acid	154.12	35.26	35.35	-0.09	154.12	0	35.16	0.1	154.12	0

NF: Not found.

Table S2. Phenolic acids found in peel and pulp extracts in negative mode.

Phenolic acid	Molecular formula	Subclass	Expected mass (m/z)	Expected RT (s)	Peel				Pulp			
					RT Observed (s)	Variation RT (s)	Observed mass (m/z)	Mass variation (m/z)	RT Observed (s)	Variation RT (s)	Observed mass (m/z)	Mass variation (m/z)
24-Methyl Cholesterol Ferulate	$C_{38}H_{56}O_4$	Hydroxycinnamic Acid	576.85	0.77	0.77	0	578.87	-2.02	NF	NF	NF	NF
Avenanthramide 2F	$C_{17}H_{15}NO_6$	Hydroxycinnamic Acid	329.3	18.42	NF	NF	NF	NF	18.67	-0.25	329.3	0
4-Glucogallic Acid	$C_{13}H_{16}O_{10}$	Hydroxybenzoic Acid	332.26	20.92	20.94	-0.02	332.25	0.01	20.95	-0.03	332.25	0.01
Galloyl Glucose	$C_{13}H_{16}O_{10}$	Hydroxybenzoic Acid	332.07	20.92	20.94	-0.02	332.25	-0.18	20.95	-0.03	332.25	-0.18
Ellagic Acid Glucoside	$C_{20}H_{16}O_{13}$	Hydroxybenzoic Acid	464.34	22.52	22.54	-0.02	464.32	0.02	22.53	-0.01	464.32	0.02
Protocatechuic Acid 4-O-Glucoside	$C_{13}H_{16}O_9$	Hydroxybenzoic Acid	316.26	22.29	22.96	-0.67	316.26	0	22.99	-0.7	316.26	0
P-Coumaroyl Tartaric Acid	$C_{13}H_{12}O_8$	Hydroxycinnamic Acid	296.23	23.55	23.6	-0.05	296.23	0	23.62	-0.07	296.23	0
Cafeoyl Tartaric Acid	$C_{13}H_{12}O_9$	Hydroxycinnamic Acid	312.23	23.85	23.91	-0.06	312.23	0	23.92	-0.07	312.23	0
4-Hydroxybenzoic Acid 4-O-Glucoside	$C_{13}H_{16}O_8$	Hydroxybenzoic Acid	300.26	25.42	25.49	-0.07	300.26	0	25.5	-0.08	300.26	0
Chicoric Acid	$C_{22}H_{18}O_{12}$	Hydroxycinnamic Acid	474.37	28.8	NF	NF	NF	NF	29	-0.2	474.37	0

NF: Not found.

Table S3. Flavonoids found in peel and pulp extracts in positive mode.

Flavonoids	Molecular formula	Subclass	Expected mass (m/z)	Expected RT (s)	Peel				Pulp			
					Observed RT (s)	Variation RT (s)	Observed mass (m/z)	Mass variation (m/z)	Observed RT (s)	Variation RT (s)	Observed mass (m/z)	Mass variation (m/z)
Butein	$C_{15}H_{12}O_5$	Chalcone	272.25	0.01	NF	NF	NF	NF	0.17	-0.16	272.26	-0.01
Naringenin	$C_{27}H_{32}O_{14}$	Flavanone	580.53	0.01	NF	NF	NF	NF	0.17	-0.16	272.26	308.27
Kaempferol	$C_{15}H_{10}O_6$	Flavonol	286.23	20.99	21	-0.01	286.24	-0.01	20.95	0.04	286.24	-0.01
Luteolin	$c_{15}h_{10}o_6$	Flavone	286.23	20.99	21	-0.01	286.24	-0.01	20.95	0.04	286.24	-0.01
Scutellarein	$C_{15}H_{10}O_6$	Flavone	286.24	20.99	21	-0.01	286.24	0	20.95	0.04	286.24	0
Cyanidin	$C_{15}H_{11}O_6$	Anthocyanins	287.24	21	21.03	-0.03	287.24	0	20.94	0.06	287.24	0
6-Hydroxyluteolin	$C_{15}H_{10}O_7$	Flavone	302.23	21.13	21.15	-0.02	302.23	0	21.01	0.12	302.23	0
Pelargonidin	$C_{15}H_{11}O_5$	Anthocyanin	271.24	22.15	22.03	0.12	271.24	0	22.92	-0.77	271.24	0
Isorhamnetin	$C_{16}H_{12}O_7$	Flavonols	316.26	22.93	23.11	-0.18	316.26	0	22.94	-0.01	316.26	0
Nepetin	$C_{16}H_{12}O_7$	Flavone	316.26	22.93	23.11	-0.18	316.26	0	22.94	-0.01	316.26	0
Ramnetin	$C_{16}H_{12}O_7$	Flavonols	316.26	22.93	23.11	-0.18	316.26	0	22.94	-0.01	316.26	0
Dihydroquercetin	$C_{15}H_{12}O_7$	Dihydroflavonol	304.25	23.78	23.69	0.09	304.25	0	23.55	0.23	304.25	0
Daidzin	$C_{21}H_{20}O_9$	Isoflavonoid	416.38	24.32	24.25	0.07	416.37	0.01	24.27	0.05	416.37	0.01
Eriodictyol	$C_{15}H_{12}O_6$	Flavanone	288.25	24.96	24.77	0.19	288.25	0	25	-0.04	288.24	0.01
Sakuranetin	$C_{16}H_{14}O_5$	Flavanone	286.27	25.26	25.46	-0.2	286.27	0	25.44	-0.18	286.27	0
Hispidulin	$C_{16}H_{12}O_6$	Flavone	300.26	25.42	25.66	-0.24	300.26	0	25.65	-0.23	300.26	0

Peonidin	$C_{16}H_{13}O_6$	Anthocyanin	301.27	25.42	25.41	0.01	301.27	0	25.43	-0.01	301.27	0
Kaempferide	$C_{16}H_{11}O_6$	Flavonols	299.25	25.43	25.5	-0.07	299.26	-0.01	25.47	-0.04	299.26	-0.01
Pelargonidin 3-O-Arabinoside	$C_{20}H_{19}O_9$	Anthocyanin	403.36	25.52	25.37	0.15	403.36	0	25.39	0.13	403.36	0
Kaempferol 3-O-Glucuronide	$C_{21}H_{18}O_{12}$	Flavonols	462.36	25.54	25.37	0.17	462.37	-0.01	NF	NF	NF	NF
Luteolin 7-O-Glucuronide	$C_{21}H_{18}O_{12}$	Flavone	462.36	25.54	25.37	0.17	462.37	-0.01	NF	NF	NF	NF
Myricetin	$C_{15}H_{10}O_8$	Flavonols	318.24	26.59	26.67	-0.08	318.23	0.01	26.5	0.09	318.23	0.01
Delphinidin 3-O-Arabinoside	$C_{20}H_{19}O_{11}$	Anthocyanin	435.36	26.93	26.89	0.04	435.35	0.01	26.72	0.21	435.35	0.01
Biochanin A	$C_{16}H_{12}O_5$	Isoflavonoid	284.26	27.17	27.35	-0.18	284.27	-0.01	27.3	-0.13	284.27	-0.01
Geraldone	$C_{16}H_{12}O_5$	Flavone	284.26	27.17	27.35	-0.18	284.27	-0.01	27.3	-0.13	284.27	-0.01
Glycitein	$C_{16}H_{12}O_5$	Isoflavonoid	284.26	27.17	27.35	-0.18	284.27	-0.01	27.3	-0.13	284.27	-0.01
Metilgalangina	$C_{16}H_{12}O_5$	Flavonols	284.26	27.17	27.35	-0.18	284.27	-0.01	27.3	-0.13	284.27	-0.01
Morin	$C_{15}H_{10}O_7$	Flavonols	302.24	27.38	27.43	-0.05	302.23	0.01	27.44	-0.06	302.23	0.01
6''-O-Acetylglycitin	$C_{24}H_{24}O_{11}$	Isoflavonoid	488.44	27.97	27.76	0.21	488.44	0	NF	NF	NF	NF
Cyanidin 3-O-(6''-Succinyl-Glucoside)	$C_{25}H_{25}O_{14}$	Anthocyanin	549.46	28.24	28.07	0.17	549.45	0.01	NF	NF	NF	NF

NF: Not found.

Table S4. Flavonoids found in peel and pulp extracts in negative mode.

Flavonoids	Molecular formula	Subclass	Expected mass (m/z)	Expected RT (s)	Peel				Pulp			
					Observed RT (s)	Variation RT (s)	Observed mass (m/z)	Mass variation (m/z)	Observed RT (s)	Variation RT (s)	Observed mass (m/z)	Mass variation (m/z)
Kaempferol	$C_{15}H_{10}O_6$	Flavonols	286.23	20.99	21.02	-0.03	286.24	-0.01	21.03	-0.04	286.24	-0.01
Luteolin	$C_{15}H_{10}O_6$	Flavone	286.23	20.99	21.02	-0.03	286.24	-0.01	21.03	-0.04	286.24	-0.01
Scutellarein	$C_{15}H_{10}O_6$	Flavone	286.24	20.99	21.02	-0.03	286.24	0	21.03	-0.04	286.24	0
Cyanidin	$C_{15}H_{11}O_6$	Anthocyanins	287.24	21	21.02	-0.02	287.24	0	21.03	-0.03	287.24	0
6-Hydroxyluteolin	$C_{15}H_{10}O_7$	Flavone	302.23	21.13	20.92	0.21	302.24	-0.01	21.2	-0.07	302.24	-0.01
Apigenin 7-O-Glucuronide	$C_{21}H_{18}O_{11}$	Flavone	446.4	22.24	22.28	-0.04	446.36	0.04	22.3	-0.06	446.36	0.04
Cirsimaritin	$C_{17}H_{14}O_6$	Flavone	314.29	22.64	22.67	-0.03	314.29	0	22.69	-0.05	314.29	0
Isorhamnetin	$C_{16}H_{12}O_7$	Flavonols	316.26	22.29	22.96	-0.67	316.26	0	22.99	-0.7	316.26	0
Nepetin	C16H12O7	Flavone	316.26	22.29	22.96	-0.67	316.26	0	22.99	-0.7	316.26	0
Ramnetin	$C_{16}H_{12}O_7$	Flavonols	316.26	22.29	22.96	-0.67	316.26	0	22.99	-0.7	316.26	0
Daidzin	$C_{21}H_{20}O_9$	Isoflavonoid	416.38	24.32	24.32	0	416.38	0	24.31	0.01	416.38	0
Hispidulin	$C_{16}H_{12}O_6$	Flavone	300.26	25.42	25.49	-0.07	300.26	0	25.5	-0.08	300.26	0
Peonidin	$C_{16}H_{13}O_6$	Anthocyanins	301.27	25.42	25.49	-0.07	301.27	0	25.5	-0.08	301.27	0
Kaempferide	$C_{16}H_{11}O_6$	Flavonols	299.25	25.43	25.49	-0.06	299.26	-0.01	25.5	-0.07	299.26	-0.01

Kaempferol 3-O-Glucuronide	$C_{21}H_{18}O_{12}$	Flavonols	462.36	25.54	25.64	-0.1	462.36	0	25.63	-0.09	462.36	0
Luteolin 7-O-Glucuronide	$C_{21}H_{18}O_{12}$	Flavone	462.36	25.54	25.64	-0.1	462.36	0	25.63	-0.09	462.36	0
Isoxanthohumol	$C_{21}H_{22}O_5$	Flavanone	354.39	25.83	26.07	-0.24	354.39	0	NF	NF	NF	NF
Xanthohumol	$C_{21}H_{22}O_5$	Chalcone	354.39	25.83	26.07	-0.24	354.39	0	NF	NF	NF	NF
Morin	$C_{15}H_{10}O_7$	Flavonols	302.24	27.38	27.46	-0.08	302.22	0.02	NF	NF	NF	NF

NF: Not found.

Table S5. Stilbenes found in peel and pulp extracts in positive mode.

Stilbene	Molecular formula	Subclass	Expected mass (m/z)	Expected RT (s)	Peel				Pulp			
					Observed RT (s)	Variation RT (s)	Observed mass (m/z)	Mass variation (m/z)	Observed RT (s)	Variation RT (s)	Observed mass (m/z)	Mass variation (m/z)
Resveratrol	$C_{14}H_{12}O_3$	Stilbene	228.24	23.83	23.65	0.18	228.23	0.01	23.71	0.12	228.23	0.01

Table S6. Other polyphenols found in peel and pulp extracts in positive mode.

Other polyphenols	Molecular formula	Subclass	Expected mass (m/z)	Expected RT (s)	Peel				Pulp			
					Observed RT (s)	Variation RT (s)	Observed mass (m/z)	Mass variation (m/z)	Observed RT (s)	Variation RT (s)	Observed mass (m/z)	Mass variation (m/z)
Pyrogallol	$C_6H_6O_3$	Other polyphenols	126.11	1.05	1.19	-0.14	126.11	0	1.01	0.04	126.11	0

3,4-Dihydroxyphenylglycol	$C_8H_{10}O_4$	Other polyphenols	170.16	12.87	12.81	0.06	170.17	-0.01	13.05	-0.18	170.17	-0.01
Syringaldehyde	$C_9H_{10}O_4$	Hydroxybenzaldehydes	182.17	18.69	18.79	-0.1	182.16	0.01	18.53	0.16	182.16	0.01
4-Hydroxycoumarin	$C_9H_6O_3$	Hydroxycoumarin	162.44	21.13	21.05	0.08	162.14	0.3	20.96	0.17	162.14	0.3
Protocatechuic Aldehyde	$C_7H_6O_3$	Hydroxybenzaldehydes	138.12	23.56	23.54	0.02	138.13	-0.01	23.48	0.08	138.13	-0.01
1,4-Naphthoquinone	$C_{10}H_6O_2$	Naphthoquinone	158.15	23.83	23.6	0.23	158.15	0	23.67	0.16	158.15	0
3-Methylcatechol	$C_7H_8O_2$	Alkylphenols	124.14	24.89	24.81	0.08	124.12	0.02	24.95	-0.06	124.12	0.02
4-Methylcatechol	$C_7H_8O_2$	Alkylphenols	124.14	24.89	24.81	0.08	124.12	0.02	24.95	-0.06	124.12	0.02
Guaiacol	$C_7H_8O_2$	Methoxyphenols	124.14	24.89	24.81	0.08	124.12	0.02	24.95	-0.06	124.12	0.02
Phlorin	$C_{12}H_{16}O_8$	Other polyphenols	288.25	24.96	24.77	0.19	288.25	0	25	-0.04	288.24	0.01
Esculetin	$C_9H_6O_4$	Hydroxycoumarin	178.14	25	24.89	0.11	178.13	0.01	NF	NF	NF	NF
Catechol	$C_6H_6O_2$	Other polyphenols	110.11	26.13	26.22	-0.09	110.1	0.01	26.08	0.05	110.1	0.01
Esculina	$C_{15}H_{16}O_9$	Hydroxycoumarin	340.28	26.37	26.45	-0.08	340.28	0	26.44	-0.07	340.28	0
Gallic Aldehyde	$C_7H_6O_4$	Hydroxybenzaldehydes	154.12	35.26	35.35	-0.09	154.12	0	35.16	0.1	154.12	0

Isopimpinellin	$C_{13}H_{10}O_5$	Furanocoumarins	246.21	47.38	47.23	0.15	246.21	0	47.18	0.2	246.21	0

NF: Not found.

Table S7. Other polyphenols found in peel and pulp extracts in negative mode.

Other polyphenols	Molecular formula	Subclass	Expected mass (m/z)	Expected RT (s)	Peel				Pulp			
					Observed RT (s)	Variation RT (s)	Observed mass (m/z)	Mass variation (m/z)	Observed RT (s)	Variation RT (s)	Observed mass (m/z)	Mass variation (m/z)
5-Heneicosylresorcinol	$C_{27}H_{46}O_2$	Alkylphenols	402.7	0.86	0.98	-0.12	401.99	0.71	NF	NF	NF	NF
5-Nonadecenylresorcinol	$C_{25}H_{42}O_2$	Alkylphenols	473.6	18.34	18.18	0.16	373.99	99.61	18.1	0.24	373.99	99.61
Carnosic acid	$C_{20}H_{28}O_4$	Phenolic terpenes	332.43	20.89	NF	NF	NF	NF	20.95	-0.06	332.42	0.01

NF: Not found.
